# PCR-RLFP characterization of *Leishmania* spp. in domestic animals from the south-western border of Brazil

**DOI:** 10.1590/S1984-29612022035

**Published:** 2022-07-18

**Authors:** Gabriela Döwich Pradella, Taiane Acunha Escobar, Thália Pacheco dos Santos, Rammy Campos Vargas, Geórgia Camargo Góss, Patricia Aline Gröhs Ferrareze, Lívia Kmetzsch Rosa e Silva, Luísa Zuravski, Karina Braccini Pereira, Claudia Acosta Duarte, Irina Lübeck

**Affiliations:** 1 Universidade Federal do Pampa – UNIPAMPA, Uruguaiana, RS, Brasil; 2 Universidade Federal de Ciências da Saúde de Porto Alegre – UFCSPA, Porto Alegre, RS, Brasil; 3 Universidade Federal do Rio Grande do Sul ­– UFRGS, Porto Alegre, RS, Brasil

**Keywords:** Canine, diagnosis, feline, Leishmaniasis, molecular biology, Caninos, diagnóstico, felinos, Leishmaniose, biologia molecular

## Abstract

The aim of this study was to characterize *Leishmania* spp. from canine and feline samples using Polymerase Chain Reaction (PCR)- Restriction Fragment Length Polymorphism (RFLP). It was conducted in the southern region of Brazil, located at border crossings to Argentina and Uruguay. Samples were collected from 116 dogs (*Canis lupus familiaris*) and 89 cats (*Felis catus*). The PCR was performed to screen for an LT1 fragment from kinetoplast DNA (kDNA) target gene, and positive samples were subjected to a second PCR for an internal transcribed spacers (ITS1) region from ribosomal DNA (rDNA) target. RFLP was performed using the *Haemophilus aegyptius* (HAE III) restriction endonuclease (Fermentas ®). Positive samples by PCR ITS1 were sequenced and deposited in NCBI GenBank, and a phylogenetic analysis was developed. We found that 12.9% (15/116) of the samples from dogs were positive. All the 89 cat samples were negative. Positive samples were tested against *Leishmania* reference strains presenting different patterns in PCR-RFLP, and these samples showed bands denoting similarity to the standard species of *Leishmania infantum*, proven through sequencing and phylogenetic analysis. The RFLP technique, alone, was shown to be feasible for practical application and confirmation of the involved *Leishmania* spp.

## Resumo

O objetivo deste trabalho foi caracterizar espécies de *Leishmania* em amostras de caninos e felinos, utilizando-se a reação em cadeia da polimerase (PCR)- polimorfismo de comprimento de fragmento de restrição (RFLP). O estudo foi realizado na região de fronteira no sul do Brasil, divisa com Argentina e Uruguai. Amostras foram coletadas de 116 cães (*Canis lupus familiaris*) e 89 gatos (*Felis catus*). A PCR foi realizada com o gene alvo do fragmento LT1 do DNA do cinetoplasto (kDNA) para triagem e, as amostras positivas foram submetidas a uma segunda PCR com alvo ITS1 no DNA ribossomal (rDNA). O RFLP foi realizado com a endonuclease de restrição *Haemophilus aegyptius* (HAE III) (Fermentas ®). As sequências positivas no PCR-ITS1 foram depositadas no NCBI GenBank, além disso, a análise filogenética foi realizada. Foram detectadas 12,9% (15/116) amostras positivas em cães. Das 89 amostras de gatos todas foram negativas. Cepas de referência de *Leishmania*, com padrões diferentes na PCR-RFLP, e amostras positivas apresentaram similaridade das bandas com as espécies padrão de *Leishmania infantum,* o que foi comprovado no sequenciamento e análise filogenética. A técnica de RFLP, sozinha, demonstrou viabilidade para a aplicação prática e a confirmação das espécies de *Leishmania* spp. envolvidas.

## Introduction

Visceral leishmaniasis (VL), a zoonotic infectious disease, is widespread worldwide. In the past, VL was characterized as a rural zoonosis; however, it has spread into urban areas and has become a serious public health problem (WHO, 2019; PAHO, 2020).

In Brazil, VL is present in all regions and causes major health problems (Azevedo et al., 2019). The disease has been endemic in the state of Rio Grande do Sul since 2008, when the first dog was diagnosed with canine VL (CVL). At that time, when cases of both CVL and human VL (HVL) arose in this state, a serological survey was carried out among the dogs by the public healthcare service. From this, canine infection by *Leishmania infantum* was confirmed through the multilocus enzyme electrophoresis (MLEE), by the National Reference Laboratory for Leishmania Typing (LRNTL) (Massia et al., 2016) and this region was classified as a transmission area for CVL (SES, 2014; CEVS, 2021).

Since 2017, border areas have been receiving special attention from the Pan-American Health Organization, especially about the epidemiological aspects of leishmaniasis, since many countries share cases, environments and parasite, vector and reservoir species (PAHO, 2020). This situation also occurs in the cross-border region between southwestern Brazil, Argentina and Uruguay, given that the presence of HVL and CVL caused by specie(s) of the *Leishmania donovani* complex has already been reported in Brazil’s neighbors (Salomón et al., 2008, 2011; Satragno et al., 2017).

It is known that dogs are the main reservoir and domestic host in the transmission cycle and have a fundamental role in the spread of the disease in endemic areas (Otranto et al., 2017). Complementarily, infection has also been reported in other mammals that cohabit spaces where parasitized dogs are present. In this context, there have also been several reports of *L. infantum* infection in cats around the world, including in Brazil (Metzdorf et al., 2017). Moreover, da Silva et al. (2010) confirmed the first report of experimental transmission of *Leishmania infantum* from a domestic cat with feline leishmaniosis (FL) to the vector *Lutzomyia longipalpis*. This highlights the fact that the presence of infected cats in endemic areas should not be neglected.

Characterization of *Leishmania* spp. in animal infections is important, considering that different species may require distinct treatment regimens and may also have very different prognoses. Moreover, such information is also valuable in epidemiological studies, for which knowledge of the distribution of *Leishmania* spp. in human and animal hosts, and in insect vectors, is a prerequisite for designing appropriate control measures. Thus, we aimed to evaluate the RFLP technique to differentiate circulating *Leishmania* specie in samples from dogs and cats, in a newly confirmed transmission area for VL in Brazil, in the southwestern cross-border region of the state of Rio Grande do Sul.

## Materials and Methods

### Study area

This study was conducted in two border municipalities in southwestern Brazil. The municipality of Uruguaiana (29°46'55” S and 57°02'18” W) is at the western extremity of the state of Rio Grande do Sul, along the river border with Argentina, and has the largest dry port in Latin America. This municipality has an area of 5,716 km^2^, 125,435 inhabitants and a demographic density of 21.95 inhabitants/km^2^. It is a municipality with a mostly rural economy, in which rice monoculture and breeding of cattle, sheep and horses can be highlighted (IBGE, 2017, 2020b). The municipality of Barra do Quaraí (30°11' 59” S, 57°31'12” W) is the southerly neighbor of Uruguaiana and is located bordering the Uruguayan municipality of Bella Unión. It has an area of 1,056 km^2^, 4,012 inhabitants and a demographic density of 3.80 inhabitants/km^2^ (IBGE, 2020a).

The analyses for this study were developed at the Laboratory of Animal Infectious Diseases of the Federal University of Pampa (Unipampa), in Uruguaiana; and at the Laboratory of Computational Biology and Bioinformatics of the Federal University of Health Sciences (UFCSPA), in Porto Alegre.

### Sample collection

The study was approved by the Ethics Committee on Animal Experimentation of the Federal University of Pampa, under protocol numbers 22/2017 and 14/2020 and was conducted in compliance with the Brazilian national guidelines on animal experimentation.

Collected samples at the Veterinary Hospital of the Federal University of Pampa and at animal owners’ homes in the municipalities of Uruguaiana and Barra do Quaraí, between July 2018 and March 2020, were used. A total of 116 dogs (*Canis lupus familiaris*) and 89 cats (*Felis catus*) of mixed breeds and both sexes were included in this study, according to the availability of the sample for carrying out the tests and evaluate the RFLP technique. At the sample collection time, an rK39 dipstick strip kit (Dual Path Platform Rapid Test, Bio-Manguinhos^®^) was used to identify the antibody response against *Leishmania,* as a screening test (Brasil, 2011).

Analytical techniques were performed according to the type of biological sample that was available. The samples from dogs included whole blood in ethylenediamine tetraacetic acid (EDTA) tubes (n = 109), serum (n = 51), exfoliative epithelial cells from eye conjunctiva (n = 63), lymph node fine needle aspiration cytology (FNAC) (n = 19) and skin lesion samples (n = 3) (Table S1). The samples from cats, consisted of whole blood collected from the jugular or cephalic vein (n = 89) and exfoliative epithelial cells from left eye conjunctiva (n = 41) (Table S2).

### *Leishmania* reference strains

*Leishmania* promastigote reference strains from the species *L. amazonensis, L. braziliensis, L. donovani*, *L. infantum* and *L. major* (Table 1) were kindly provided by the *Leishmania* Collection of the Oswaldo Cruz Institute (CLIOC - FIOCRUZ/RJ). These were used as positive controls for the polymerase chain reaction (PCR) and for the molecular characterization technique of restriction fragment length polymorphism (RFLP).

**Table 1:** *Leishmania* strains used as reference patterns, identification of the strains and species and international reference code.

**Identification**	**Species**	**International Code**
Reference Strain 1 (Ref 1)	*L. (L.) donovani*	MHOM/ET/1967/HU3
Reference Strain 2 (Ref 2)	*L. (V.) braziliensis*	MHOM/BR/1975/M2903
Reference Strain 3 (Ref 3)	*L. (L.) amazonensis*	IFLA/BR/1967/PH8
Reference Strain 4 (Ref 4)	*L. (L.) infantum*	MHOM/BR/1974/PP75
Reference Strain 5 (Ref 5)	*L. (L.) infantum*	MHOM/BR/2002/LPC-RPV
Reference Strain 6 (Ref 6)	*L. (L.) major*	MHOM/SU/1973/5-ASKH

### DNA purification

DNA from animal samples (whole blood; exfoliative epithelial cells from eye conjunctiva; lymph node fine-needle cytological and skin lesion) and *Leishmania* reference strains was isolated using the DNeasy^®^ Blood & Tissue kit (QIAGEN^®^), following the manufacturer’s instructions for each kind of sample. The purified DNA samples were eluted in the elution buffer (TE). Electrophoresis on 1.5% agarose gel was used to verify DNA integrity, and DNA concentrations were estimated by means of the NanoVue spectrophotometer. All the samples were purified and stored at -20 °C until use.

### Polymerase Chain Reaction (PCR)

PCR amplifications were performed using two target genes separately. Firstly, all the 116 dog and 89 cat samples were tested using a primer pair from a 145-bp target sequence of the LT1 fragment, in the kDNA minicircles of the *L. donovani* complex (Le Fichoux et al., 1999). The primers used were RV1 (5’-CTTTTCTGGTCCCGCGGGTAGG-3’) and RV2 (3’-CCACCTGGGCTATTTTACACCA–5’).

Following this, the samples that were found to be positive through kDNA-PCR were subjected to a second PCR with ITS1(internal transcribed spacers) as a target gene (El Tai et al., 2001). A fragment of 320 bp was obtained through amplification using the following primer pair: LITSR (5’-CTGGATCATTT-TCCGATG-3’) and L5.8S (3’-TGATACCACTTATCGCACTT-5’).

The PCR conditions were as described elsewhere (El Tai et al., 2001; Le Fichoux et al., 1999). The reaction mixtures were adjusted to a final volume of 25 µL, and consisted of 25‐50 ng of DNA, 2.5 U of Taq DNA polymerase, 1X PCR buffer, 1.5 mM of MgCl_2_, (Invitrogen, USA), 10 mM of each dNTP (dATP, dCTP, dGTP and dTTP) (Promega, USA),10 pmol of each primer (IDT, USA) and ultrapure water. The reactions were performed in a thermocycler (Amplitherm Thermal Cyclers, USA). All assays included negative PCR controls (PCR mix without DNA), and positive PCR controls (DNA extracted from *L. infantum* promastigotes [MHOM/BR/1974/PP75]). The amplification products were viewed by means of electrophoresis on 1.5% agarose gel in 1 × TAE‐buffer, stained with ethidium bromide.

Samples that were shown to be positive through the RV1/RV2 primer pair but which were negative through ITS1 were subjected to a nested ITS1-PCR technique, as described previously (Schönian et al., 2003). In short, 2 µL of the ITS1 reaction was used in a new mix, under the same conditions as in the normal PCR described above.

### Restriction Fragment Length Polymorphism (RFLP)

RFLP analysis was performed on the PCR-amplified ITS1 gene using the HAE III (*Haemophilus aegyptius*) restriction endonuclease (Fermentas^®^, USA). This is a standardized molecular method that has been implemented for endemic areas by the Leishmaniasis Epidemiology Network South America (LeishEpiNetSA) (Cupolillo et al., 2009). After the conditions had been optimized, digestion reactions were carried out in a final volume of 25 μL, comprising 20 μL of ITS1-PCR product, 10 U of restriction endonuclease (HAEIII), 10× recommended buffer and 1.5 μL of milli-q water. All restriction reactions were incubated at 37 ºC for two hours. Afterwards, the restriction fragments were resolved by means of electrophoresis on 2% agarose gel containing SYBR green (0.05 μL/mL) in an electrophoresis cube at 90 V for 90 minutes, and were viewed using ultraviolet light (Cupolillo et al., 2009).

The amplicons were observed using a 50 bp DNA ladder (Ludwig Biotec^®^, Brazil) to define the characteristics of the *Leishmania* that was present. The reference strains were also subjected to PCR-RFLP for comparison with the samples analyzed.

### Sequencing

ITS1-PCR amplicons from 9 dogs positive samples (2 whole blood; 1 skin lesion; 1 eye conjunctiva and 5 lymph node fine-needle cytological) were confirmed by means of direct sequencing, performed in an automated sequencer ABI-Prism 3500 Genetic Analyzer (Applied Biosystems, USA). From 11 ITS1-PCR positive samples, 2 were insufficient to perform the analysis, C32 and C92. The products were then purified using the PCR Products Purification Kit (Mebep Bio Science^®^, China) in accordance with the manufacturer's instructions and were quantified using NanoVue (GE Healthcare^®^, USA). A consensus sequence for each sample was generated using the UGENE software (Okonechnikov et al., 2012), followed by manual curation for correction of dubious positions by means of sequence comparison in all sequenced fragments. The sequences AJ634355.1 and AJ634346.1 of *L. infantum* from NCBI were used as references for sequence alignment by means of the MAFFT v.7 aligner (Katoh et al., 2019). The ITS1 gene sequences were deposited in NCBI GenBank.

### Phylogenetic analysis

The phylogenetic analysis was performed using 29 sequences; 9 of them were from this study (C06, C07, C97, C99, C104, C110, C111, C113 and C114), 2 ITS1-PCR sequences were insufficient to perform the analysis (C32 and C92). The sequence alignment was generated by the MAFFT v.7 web server (1PAM / κ = 2 scoring matrix) (Katoh et al., 2019), while the alignment trimming of the 5’ and 3’ ends was performed using UGENE, thus generating an alignment size of 494 characters. The best evolutionary model was inferred to be TPM3uf+G4, by ModelTest-ng (Darriba et al., 2020).

The first phylogenetic tree was built by means of the maximum likelihood (ML) method, using the IQ-TREE v1.6.1 software (Nguyen et al., 2015) with a Shimodaira-Hasegawa-like approximate likelihood ratio test (SH-aLRT) of 1,000 replicates (Guindon et al., 2010). This was added to an ultrafast bootstrap of 1,000 replicates, and the UFBoot trees were optimized by means of NNI on bootstrap alignment (Hoang et al., 2018).

The second phylogenetic tree was built by means of Bayesian inference using the MrBayes v3.2.7 software (Huelsenbeck & Ronquist, 2001) and GTR+G4 (nst = 6, rates = gamma) as an evolutionary model in substitution for TPM3uf+G4. For MrBayes analyses, 3,000,000 generations were computed; the MCMC convergence was evaluated using the Tracer v.1.7.1. software (Rambaut et al., 2018). The tree visualization and editing were generated using the FigTree software (http://tree.bio.ed.ac.uk/software/figtree/). The cutoff for clade definition was set using branch support values ≥ 80% for SH-aLRT and ultrafast bootstrap tests.

## Results

The screening test (DPP), performed on all the 116 canine samples, resulted in 37% (43/116) positive animals. We found that 12.9% (15/116) of the samples from dogs in urban areas were positive according to PCR using the primers RV1/RV2, of these, 13 were also positive in the rapid test. Among these 15 PCR-positive samples we found fragments (RV1/RV2 primers) of *Leishmania* spp. from whole blood (n = 7), lymph node fine-needle cytological samples (n = 5), skin lesion samples (n = 2) and conjunctival samples (n = 1). The kind of sample extracted from each animal could be visualized in Table S2. ITS1-PCR was performed on all kDNA-positive dog samples (n=15) to identify *Leishmania* spp., and 11 samples (3 whole blood; 2 skin lesions, 1 eye conjunctival and 5 lymph node fine-needle cytological) were shown to be positive, using nested PCR when necessary (Table 2). No genetic material from the parasite was detected in any of the samples from cats that were cohabiting with dogs, these samples will be subjected to further analysis.

**Table 2:** Dogs that were positive through the PCR technique, with their identifications, sex, age, DPP^®^ rapid test results, sample source and PCR RV1/RV2 and PCR ITS1 results.

**Animal**	**Sex**	**Age**	**DPP**	**Sample**	**PCR RV1/RV2**	**PCR ITS1**
C06	Male	3	**+**	whole blood	**+**	**+**
C07	Male	6	**+**	whole blood	**+**	**+**
C09	Female	-	**+**	whole blood	**+**	**-**
C32	Female	-	**+**	whole blood	**+**	**+**
C43	Male	8	**+**	whole blood	**+**	**-**
C45	Female	5	**-**	whole blood	**+**	**-**
C70	Female	10	**+**	whole blood	**+**	**-**
C92	Female	3	**+**	skin lesions	**+**	**+**
C97	Female	7	**+**	skin lesions	**+**	**+**
C99	Male	10	**+**	conjunctival	**+**	**+**
C104	Female	12	**+**	Fine needle aspiration cytology	**+**	**+**
C110	Male	4	**+**	Fine needle aspiration cytology	**+**	**+**
C111	Female	2	**+**	Fine needle aspiration cytology	**+**	**+**
C113	Female	-	**-**	Fine needle aspiration cytology	**+**	**+**
C114	Female	2	**+**	Fine needle aspiration cytology	**+**	**+**

**Figure 1:**
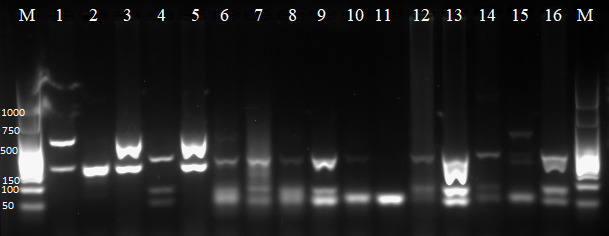
Presentation of DNA bands on agarose gel, from standard *Leishmania* strains and from samples from positive dogs: *L. donovani* (1); *L. braziliensis* (2); *L. amazonensis* (3); *L. infantum* (4); *L. major* (5); C06 (6); C07 (7); C32 (8); C92 (9); C97 (10); C99 (11); C104 (12); C110 (13); C111 (14); C113 (15); C114 (16); and 50 bp DNA ladder (M).

Species identification by means of PCR-RFLP was performed on ITS1-PCR positive samples from 11 dogs and on *Leishmania* reference strains. As expected, the *Leishmania* reference strains showed different patterns in PCR-RFLP. The species *L. amazonensis, L. donovani* and *L. major* presented two bands in agarose gel. On the other hand, *L. infantum* showed three bands, among which two were between 50 and 100 bp in size. *L. braziliensis* showed two bands close to 150 bp. Positive samples from PCR-RFLP showed the same band characteristics as the *L. infantum* control. Three samples presented different patterns that diverged from the species used as references. However, more similarity with *L*. *infantum* was seen than with other species (Figure 1). Despite that, the sequencing confirmed *L. infantum*.

Among the 11 samples that were subjected to the RFLP technique, it was possible to perform sequencing on 9 of them, because 2 did not offer enough material (C32 and C92). The consensus sequence generated using the UGENE software (Okonechnikov et al., 2012), followed by manual curation, is illustrated in Table 3. After analysis on GenBank data, the samples from these 9 dogs confirmed the species as *L. infantum* (Table 4).

**Table 3:** *Leishmania* DNA sequences from clinical samples from dogs.

**Sequence ID**	**Alignment**
C06	AATTCTGGATCATTTTCSGATGATTACACCCAAAAAACATATACAACTCGGGGAGACCTATGTATATATATGTAGGCCTTTCCCACATACACAGCAAAGTTTTGTACTCAAAATTTGCAGTAAAAAAAAGGCCGATCGACGTTATAACGCACCGCCTAAACAAAAGCAAAAATGTCCGTTTATACAAAAAATATACGGCGTTTCKGTTTTTGGCGGGGTGGGTGCGTGTGTGGATAACGGCTCACATAACGTGTCGCGATGGATGACTTGRCTTCCTATTTCGTTGAAGAACGCASTAAAGTGCCATAAGTGGTATCAA
C07	TCTGGATCATTTTCCGATGATTACACCCAAAAAACATATACAACTCGGGGAGACCTATGTATATATATGTAGGCCTTTCCCACATACACAGCAAAGTTTTGTACTCAAAATTTGCAGTAAAAAAAAGGCCGATCGACGTTATAACGCACCGCCTATACAAAAGCAAAAATGTCCGTTTATACAAAAAATATACGGCGTTTCGGTTTTTGGCGGGGTGGGTGCGTGTGTGGATAACGGCTCACATAACGTGTCGCGATGGATGACTTGGCTTCCTATTTCGTTGAAGAACGCAGTAAAGTGCGATAAGTGGTATCAA
C104	ATTCTGGATCATTTTCYGATGATTACACCCAAAAAACATATACAACTCGGGGAGACCTATGTATATATATGTAGGCCTTTCCCACATACACAGCAAAGTTTTGTACTCAAAATTTGCAGTAAAAAAAAGGCCGATGGATCGTTATAACGCACCGCCTWAATACTGTGTTCAAAGTCCCGTTTATGCTATCAATATMCGGCGTTTCKGTTTTTGGCGGGGTGGGTGCGTGTGTGGATAACGGCTCACATAACGTGTCGCGATGGATGACTTGGCTTCCTATTTCSTTGAAGAACGCASTAAAKTGCGATAAGTGGKATCAACAC
C110	TTCTGGATCATTTTCCGATGATTACACCCAAAAAACATATACAACTCGGGGAGACCTATGTATATATATGTAGGCCTTTCCCACATACACAGCAAAGTTTTGTACTCAAAATTTGCAGTAAAAAAAAGGCCGATCGACGTTATAACGCACCGCCTATACAAAAGCAAAAATGTCCGTTTATACAAAAAATATACGGCGTTTCGGTTTTTGGCGGGGTGGGTGCGTGTGTGGATAACGGCTCACATAACGTGTCGCGATGGATGACTTGGCTTCCTATTTCGTTGAAGAACGCAGTAAAGTGCGATAAGTGGTATCAAAAAT
C111	TCTGGATCATTTTCCGATGATTACACCCAAAAAACATATACAACTCGGGGAGACCTATGTATATATATGTAGGCCTTTCCCACATACACAGCAAAGTTTTGTACTCAAAATTTGCAGTAAAAAAAAGGCCGATCGACGTTATAACGCACCGCCTATACAAAAGCAAAAATGTCCGTTTATACAAAAAATATACGGCGTTTCGGTTTTTGGCGGGGTGGGTGCGTGTGTGGATAACGGCTCACATAACGTGTCGCGATGGATGACTTGGCTTCCTATTTCGTTGAARAACGCAGTAAAGTGCGATAAGTGGTATCA
C113	CTTCTGGATCATTTTYYGATGATTACACCCAAAAAACATATACAAMTCGGGGAGACCTATGTATATATATGTAGGCCTTTCCCACATACACAGCAAAGTTTTGTACTCAAAATTTGCAGTAAAAAAAAGGCCGATGGATCGTTATAACGCACCGCCTATACAAAAGCCCAAAGRTCCGTTTATCGTATCAATATACGGCGTTTCGGTTTTTGGCGGGGTGGGTGCGTGTGTGATAACGGCTCACATAACGTGTCGCGATGGATGACTTGGCTTCCTATTTCGTTGAAGAACGCAATAGAGTGCGATAAGTGGTATCAA
C114	TCTGGATCATTTTCCGATGATTACACCCAAAAAACATATACAACTCGGGGAGACCTATGTATATATATGTAGGCCTTTCCCACATACACAGCAAAGTTTTGTACTCAAAATTTGCAGTAAAAAAAAGGCCGATCGACGTTATAACGCACCGCCTATACAAAAGCAAAAATGTCCGTTTATACAAAAAATATACGGCGTTTCGGTTTTTGGCGGGGTGGGTGCGTGTGTGGATAACGGCTCACATAACGTGTCGCGATGGATGACTTGGCTTCCTATTTCGTTGAAGAACGCAGTAAAGTGCGATAAGTGGTATCAA
C97	CTGGATCATTTTCCGATGATTACACCCAAAAAACATATACAACTCGGGGAGACCTATGTATATATATGTAGGCCTTTCCCACATACACAGCAAAGTTTTGTACTCAAAATTTGCAGTAAAAAAAAGGCCGATCGACGTTATAACGCACCGCCTATACAAAAGCAAAAATGTCCGTTTATACAAAAAATATACGGCGTTTCGGTTTTTGGCGGGGTGGGTGCGTGTGTGGATAACGGCTCACATAACGTGTCGCGATGGATGACTTGGCTTCCTATTTCGTTGAAGAACGCAGTAAAGTGCGATAAGTGGTATCA
C99	ATCTGGATCATTTTCCGATGATTACACCCAAAAAACATATACAACTCGGGGAGACCTATGTATATATATGTAGGCCTTTCCCACATACACAGCAAAGTTTTGTACTCAAAATTTGCAGTAAAAAAAAGGCCGATGGATCGTTATAACGCACCGCCTATACAAAAGCAAAAATGTCCGTTTATGCTATAAATATACGGCGTTTCKGTTTTTGGCGGGGTGGGTGCGTGTGTGGATAACGGCTCACATAACGTGTCGCGATGGATGACTTGGCTTCCTATTTCKTTGAAGAACGCARTAAAGTGCGATAAGTGGTATCAACTACGATATCWCTACAGGCT

**Table 4:** GenBank accession number for each positive sample from dogs and information on collection date, organism identified (BLAST analysis) and host (sample source).

**Sample**	**GenBank accession number**	**Collection date**	**Organism**	**Host**
C06	MZ788622	19-Sep-2018	*Leishmania infantum*	*Canis lupus familiaris*
C07	MZ788623	26-Sep-2018	*Leishmania infantum*	*Canis lupus familiaris*
C104	MZ788624	03-Sep-2020	*Leishmania infantum*	*Canis lupus familiaris*
C110	MZ788625	08-Sep-2020	*Leishmania infantum*	*Canis lupus familiaris*
C111	MZ788626	08-Sep-2020	*Leishmania infantum*	*Canis lupus familiaris*
C113	MZ788627	09-Sep-2020	*Leishmania infantum*	*Canis lupus familiaris*
C114	MZ788628	09-Sep-2020	*Leishmania infantum*	*Canis lupus familiaris*
C97	MZ788629	04-Mar-20	*Leishmania infantum*	*Canis lupus familiaris*
C99	MZ788630	11-Mar-20	*Leishmania infantum*	*Canis lupus familiaris*

**Figure 2:**
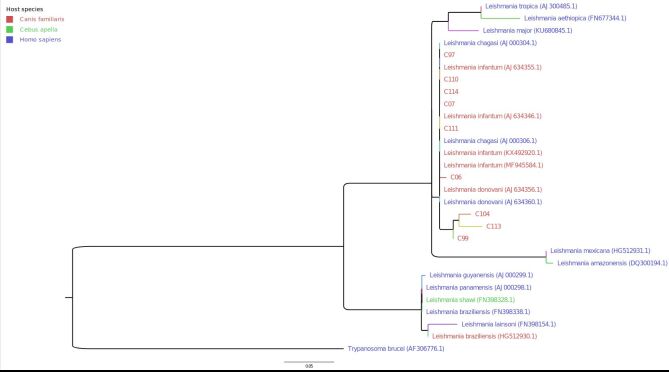
Phylogenetic tree for *Leishmania* spp. isolates from southern Brazil, generated from maximum likelihood inference analysis on kDNA sequences. The branch support values represent the results from SH-aLRT and ultrafast bootstrap tests, respectively. The sample names are colored according to the host species. The isolates are described in the Supplementary file.

Figure 2 illustrates the phylogenetic tree of *Leishmania* spp. isolates from southern Brazil. In comparison with the *Trypanosoma brucei* sequence, located at the base of the phylogenetic tree, 2 monophyletic clades are observed. For the first one, comprising all the sequenced samples from this study, as well as sequences from *L. aethiopica, L. amazonensis, L. donovani, L. infantum, L major, L. mexicana* and *L. tropica*, there was statistical support of 92.7% through the SH-aLRT test and 79% through the ultrafast bootstrap. For the second one, which included the kDNA sequences from the species *L. braziliensis, L. guyanensis, L. lainsoni, L. panamensis* and *L. shawi*, there was statistical support of 89.3% through the SH-aLRT test and 94% through the ultrafast bootstrap. Inside the first clade, formation of three internal subclades could be seen: (1) *L. aethiopica, L. major* and *L. tropica*, with branch support of 91.6/91% (SH-aLRT/ultrafast bootstrap); (2) *L. amazonensis* and *L. mexicana*, with branch support of 100% through both tests; and (3) *L. donovani*, *L. infantum*/ *L. chagasi*, i.e. the set added to the samples from this study, with branch support of 83.4% through the SH-aLRT test. Because of the high similarity between the kDNA sequences from this third subclade (C99, C104 and C113 accumulated the highest divergence, forming a small monophyletic group with branch support of 97.5% through the SH-aLRT test), it was not possible to precisely define the species of these samples through phylogenetic analysis.

The sequences from *L. chagasi* (AJ000306.1 and AJ000304.1) were identical to those from *L. infantum* (AJ634346.1 and AJ634355.1), as expected, since these only differed in nomenclature. Likewise, the sequences of *L. donovani* were identical to each other (Figure 2).

Four samples were identical to *L. infantum / L. chagasi*. Among the other sequenced samples, the variability could either have been natural or due to sequencing error, since there was little basic consensus on some positions. Naturally, C99, C104 and C113 would form a separate group, with their own ancestor, compared with the others (Figure 2).

**Figure 3:**
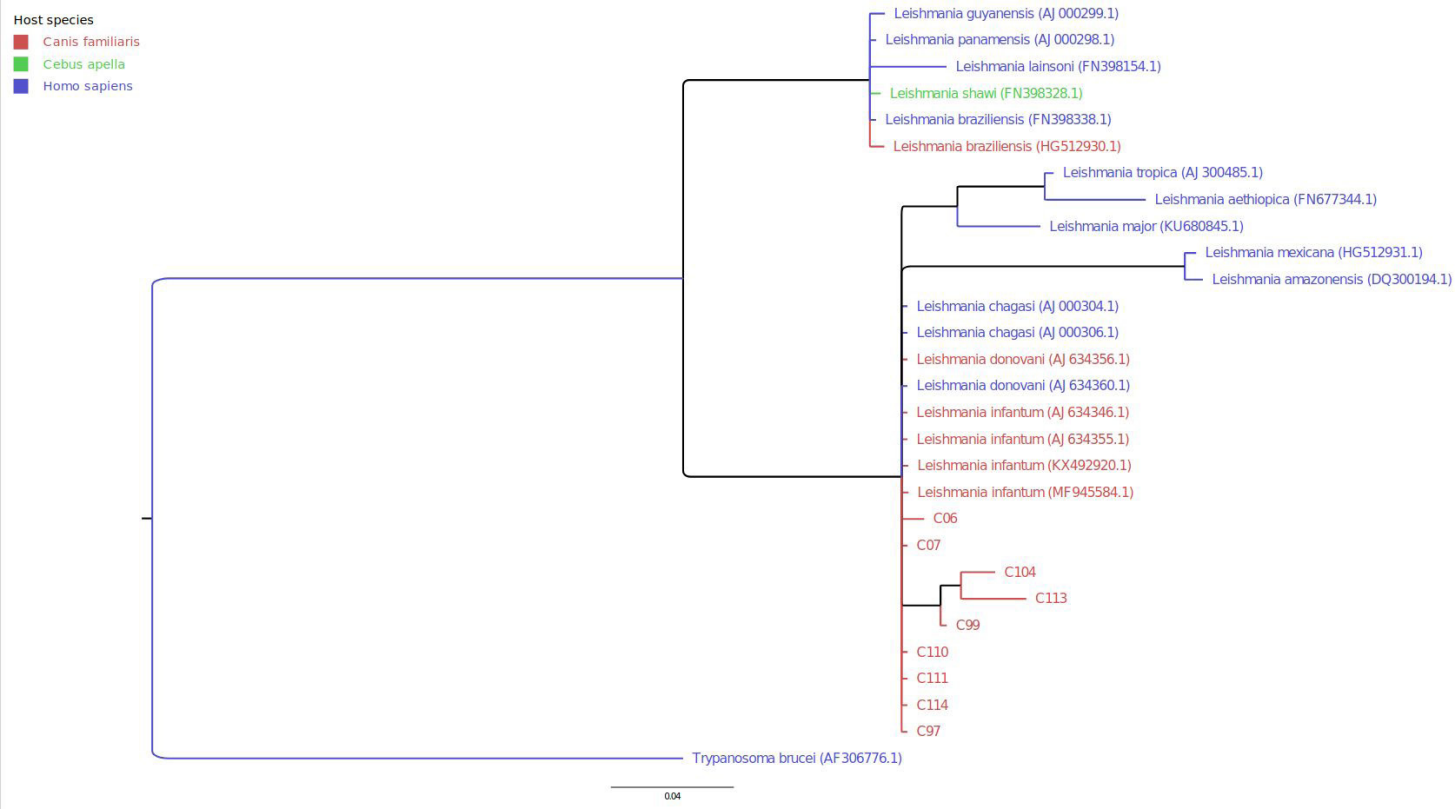
Phylogenetic tree for *Leishmania* spp. isolates from southern Brazil, generated from Bayesian inference analysis on kDNA sequences. The branch support values represent the results from posterior probabilities. The sample names are colored according to the host species. The isolates are described in the Supplementary file.

The additional phylogenetic analysis by means of Bayesian inference (Figure 3) reached similar tree topology, with samples clustering in two major monophyletic clades (branch support of 100%, according to posterior probabilities). Differently from the ML analysis, the Bayesian tree presented the species *L. donovani* and *L. infantum/ L. chagasi*, along with six samples from this study, in the basal branch of this second clade. Three internal subclades were formed: (1) *L. aethiopica, L. major* and *L. tropica* group (branch support of 98%, according to posterior probabilities); (2) *L. amazonensis* and *L. mexicana* (branch support of 100%, according to posterior probabilities); and (3) C99, C104 and C113 (from this study), with statistical support of 84%.

Through characterization of *L. infantum* in the field samples, the occurrence of CVL in this region was elucidated, and this corroborated previous studies (Escobar et al., 2019, 2020; Pradella et al., 2020). The samples allowed the RFLP evaluation and, posterior sequencing and phylogenetic tree of *Leishmania* spp. isolates reinforce the applicability of RFLP in field samples to differentiate circulating species.

## Discussion

Based on the existing evidence of vector species and the clinical manifestations and previous diagnoses among dogs and humans in this region, we characterized the circulating *Leishmania* species in a Brazilian border using PCR-RFLP. Thus, we firstly used diagnostic analysis with kDNA markers, which have higher numbers of copies per cell (~ 10,000 copies per cell). Then, among positive samples, we used a second PCR with the ribosomal internal transcribed spacer marker (40-200 copies per cell). All clinically important species could be distinguished by their RFLP patterns. ITS1 PCR with one digestion of amplicons by the restriction endonuclease (HAEIII) is sufficient to distinguish almost all medically relevant *Leishmania* spp., and thus can separate the *L. donovani* complex from other complex species (Schönian et al., 2003). To date, we have found no evidence of species that are etiological agents for cutaneous leishmaniasis in the region.

We confirmed the presence of *Leishmania infantum* DNA in dogs from south-eastern extremity of Brazil. We found 12.9% of positive PCR among the dogs through using the primers RV1/RV2, and 37% through using the rapid test DPP. In an epidemiological survey carried out between years 2009 and 2010, by the health surveillance department of Uruguaiana, samples were collected from 965 dogs and 43 (4.4%) of these were seropositive for CVL (Massia et al., 2016). This study does not aim to elucidate the disease prevalence, but the characterization of circulating *Leishmania* spp., so, the technique and samples collection was different in the studies, precluding the percentage comparison. However, both kind of evaluation are extremely required.

We did not verify any spread of the infection to cats through using a conventional PCR technique on peripheral blood samples from them. This may have been related to the technique and the type of sample used. We took conjunctival swabs from these cats, but unlike in other studies, we were not successful in extracting DNA through this collection method (Benassi et al., 2017; Costa-Val et al., 2020). Feline samples could be subjected to other diagnostic techniques, such as real-time PCR, which represents an advance in relation to classic methodologies, in terms of the possibilities of automation, high throughput, rapidity and high sensitivity (Galluzzi et al., 2018). One limitation of our survey was the volume of samples collected and the difficulty of locating these cats later, for new sample collection, considering that most of them were only semi-domiciled and had access to the streets.

Regarding molecular techniques, the PCR provides a highly specific test (Iniesta et al., 2002). A variety of primer pairs are capable of amplifying different *Leishmania* genome regions (Lachaud et al., 2002). The LITSR and L58S primer pairs amplify the ITS1 gene region (rDNA size: 320 base pairs) (El Tai et al., 2000) and, in association with the RFLP, can identify the species *L. infantum* (Schönian et al., 2003), as observed in the present study. The PCR technique followed by RFPL enabled identification of *L. infantum* in canine samples, which was later on confirmed through sequencing and construction of the phylogenetic tree.

We also observed that samples C45 and C113, positive in PCR RV1/RV2, were negative in the DPP rapid test. This may have been related to the infection phase and immune response from the dogs, in view of the methodological differences between analyses (Maciel et al., 2020). Moreover, out of the 43 samples that were positive through the DPP test, only 13 were positive through the PCR technique using RV1/RV2 primers. This can be related to the same fact.

Species identification is crucial for diagnosis, treatment, control and prevention measures. Use of molecular and biochemical tools for taxonomic classifications and phylogenetic studies on the diversity of *Leishmania* spp. around the world has been increasing. Classifications have been based on geographical distribution, vector species and disease presentations (Schönian et al., 2018). The presence of *L. infantum* in the Brazil-Argentina-Uruguay transborder region was only discovered recently: until the past decade, this infection had never been diagnosed in southern Brazil. In addition, canine and human leishmaniasis cases have now been detected, as well as infections among horses (Escobar et al., 2019; Pradella et al., 2020, 2021).

This border region was not previously included as a focus of the federal healthcare system and it was believed that the disease would be cutaneous and not visceral, as studies had shown. However, the western region of Rio Grande do Sul, located on the triple border of Brazil, Uruguay and Argentina, now forms an area of VL transmission. Since 2011, it has been classified as an area of extreme epidemiological importance (Deboni et al., 2011), with reports of the vector and infection in humans and dogs in the Corrientes province of Argentina (Berrozpe et al., 2017) and in the departments of Artigas and Salto in Uruguay (Salomón et al., 2011). It has been hypothesized that the vector may have undergone considerable adaptation along the margins of large rivers, such as the Uruguay River, with dissemination of the infection in these areas (Satragno et al., 2017).

Through this study, we have started to monitor the infective species. Our region has not been free from infection and disease for more than 12 years and thus it appears that excellent vector-host adaptation has occurred. Recognition of the present species makes it possible to develop studies to raise awareness among the population about care and prevention, as well as informing and alerting the medical and veterinary community and healthcare authorities about the occurrence of this disease and the need to investigate the infection in the population.

## Conclusions

The PCR-RFLP technique enabled differentiation of *Leishmania* strains with clinical importance, using the HAEIII restriction endonuclease. Through using PCR RV1/RV2 primers, we detected that 12.9% (15/116) of the samples were positive, among dogs in urban areas, while none of the samples from cats were positive. With regard to the dogs, 11 were also positive through ITS1 PCR, and these were subjected to the RFLP technique. In comparisons with the reference strains of *Leishmania*, the canine samples showed similarity to the *L. infantum* species. This was proven subsequently, through sequencing 9 of these samples and performing phylogenetic analysis. The present study confirmed that it was possible to diagnose CVL in this region through molecular diagnosis, and the RFLP technique was demonstrated to be feasible for practical application.

## Conflict of interest statement

The authors declare no conflict of interest.

## Supplementary material accompanies this paper.

Table S1. Canine samples

Table S2 felino

This material is available as part of the online article from https://www.scielo.br/j/rbpv
